# Knockdown of G protein-coupled receptor-17 (GPR17) facilitates the regeneration and repair of myelin sheath post-periventricular leukomalacia (PVL)

**DOI:** 10.1080/21655979.2021.1979352

**Published:** 2021-09-27

**Authors:** Liufang He, Hui Yang, Jinxing Feng, Tingyan Wei, Yong Huang, Xueli Zhang, Zhangxing Wang

**Affiliations:** a Department of Neonatology, Affiliated Longhua People’s Hospital, Southern Medical University (Longhua People’s Hospital), Shenzhen, China; b Department of Neonatology, Shenzhen Children Hospital, Shenzhen, China

**Keywords:** G protein-coupled receptor-17, periventricular leukomalacia, oligodendrocyte transcription factor-1

## Abstract

The G protein-coupled receptor-17 (GPR17) plays an important role in regulating the differentiation of oligodendrocytes and remyelination, which is a key negative regulator of oligodendrocyte differentiation. The present study aimed to investigate the function of GPR17 in the white matter of periventricular leukomalacia (PVL) neonatal rats. The PVL model was established in 2-day old neonatal rats by intracerebral injection of LPS (1 mg/kg). Compared to sham, GPR17 was significantly upregulated, while Olig1 was significantly downregulated in the PVL group at 1 d, 3 days, and 7 days post-modeling. Compared to the negative control (NC) group, the expression of GPR17 was suppressed, while that of Olig1 was elevated in the siRNA-GPR17 group as time progressed; the opposite results were observed in the GPR17-overexpressed group. Decreased formation of myelin sheaths as well as poor structure and loose arrangement were observed in the PVL group. Similar observations were found in the PVL + siRNA-GPR17 group at 1 d and 3 days post-modeling. However, on day 7 post-modeling, a dramatic increase in the formation of myelin sheath as well as thicker myelin sheaths were observed in the PVL + siRNA-GPR17 group. The migration ability of oligodendrocyte progenitor cells (OPCs) isolated from animals was found to be significantly suppressed in the GPR17-overexpressed group, accompanied by the downregulation of Olig1. Taken together, the regeneration and repair of myelin sheaths post-PVL white matter injury were induced by downregulating the GPR17 gene, which elevated the expression of Olig1.

## Introduction

Periventricular leukomalacia (PVL) is a type of brain injury commonly observed in premature infants, and it is pathologically characterized by injury and loss of oligodendrocyte (OL) precursors, which finally contribute to myelin damage and a decrease in nerve fiber myelination [1,2]. The morbidity of PVL in premature infants is approximately 26%–60%, with PVL clinically accompanied by sequelae including cerebral palsy, audiovisual and cognitive impairments, and mental retardation [3]. As neonatal intensive care units are gradually being established and developed, the rescue success rate of premature infants has increased significantly, which further results in an increase in PVL morbidities. The improvement of the quality of life of premature infants has become an international goal of neonatal medicine during this century. Currently, no effective therapy is available for the treatment of PVL. It is of great significance to investigate the pathological mechanisms and therapeutic strategies of PVL to improve premature infants’ survival rates, as well as their quality of life [4].

OL plays an important role in the formation of the myelin sheath in the central nervous system (CNS), the neurite of which wraps the nerve fiber to form a myelin sheath [5]. Myelinated axons are vital for the accelerated processing of information in the brain. The transmission rate of myelinated axons is approximately 430 km/h, while the transmission rate of non-myelinated axons is approximately 3.6 km/h [6]. If OL is damaged, severe disability will be induced by a decreased or terminated transmission rate due to a damaged myelin sheath [7]. In the white matter of the brain of human premature infants, neural progenitors generally differentiate into OL precursors, immature OLs, and mature OLs [8]. It has been reported that OL precursors are the main target cells for the development of PVL and play an important role in the pathogenesis of PVL [9]. The differentiation and remyelination of OL precursors are positively regulated by the transcription factor Olig1 [10]. Therefore, targeting Olig1 might be an effective method for the treatment of PVL by regulating the process of differentiation from neural progenitors to OL precursors.

GPR17 is mainly expressed in the brain, heart, kidney, and skeletal muscle, and is expressed at low levels in the liver and lung [11]. In the brain, GPR17 is mainly located in the white matter, gray matter, dentate gyrus, and corpus callosum [12], and it is expressed in inactivated neural progenitors and neurons [13]. When a mature OL is differentiated or myelination reaches its peak, the expression of GPR17 decreases greatly [14]. As a sensor of brain injury, GPR17 has been regarded as the key protein that regulates the endogenous repair of brain injury. Approximately 72 h after blocking the brain artery of rats, there was apparent proliferation of BrdU^+^/GPRl7^+^ neural progenitors in the marginal area of injured white matter, which was accompanied by an increase in the number of mature OLs [15].

In the present study, we hypothesize that GPR17 is involved in the pathogenesis of PVL, which is associated with the regulation of Olig1 expression. The expression and biological function of GPR17 in the white matter of PVL neonatal rats will be investigated to explore potential therapeutic targets for the treatment of clinical PVL.

## Materials and methods

### Construction of the GPR17 recombinant adenovirus vector

The siRNA targeting GPR17 was synthesized by Shanghai Genechem Co., Ltd., and the sequence was 5ʹ GCTTCGAAGACCTCTCTAT3ʹ. The two oligonucleotides were dissolved in deionized water and mixed to form double-stranded oligonucleotides after annealing. The purified pDC314 plasmids were obtained following double digestion of the siRNA double-stranded oligonucleotide using BamHI and SaII (Sigma, MA, USA), which was transfected into competent DH5α bacteria for amplification to obtain the shuttle vector of GPR17 siRNA (GV120/GPR17 siRNA) [16]. The recombinant vector was confirmed using DNA sequencing.

### Construction and identification of recombinant shuttle plasmid pDC315/GPR17

Total RNA was isolated from the brain tissue of neonatal rats using Trizol solution and were transformed into cDNAs, which were used as a template to amplify the GPR17 gene. The forward primer was 5ʹ-GCTCTTCGCCTGCTTCTACC-3ʹ, while the reverse primer was 5ʹ-GCGGACGGCTTTATTCTTGA-3ʹ. After recovery and purification, the GPR17 gene and pDC315 were double-digested. The digested target fragments were linked to the T4 DNA ligase. The linkage product was transfected into DH5α bacteria for amplification. The recombinant shuttle plasmid pDC315/GPR17 was obtained by screening, amplification, and isolation. The accuracy of the recombinant plasmids was verified by sequencing [17].

### Packaging and amplification of recombinant adenovirus

One day before transfection, HEK293 cells, which were purchased from ATCC (ATCC, USA), were seeded on 6-well plates at a density of 1 × 10^6^ cells/well and incubated with DMEM complete medium containing 10% fetal bovine serum at 37°C and 5% CO_2_ for 12–24 hours until 70%–80% cells were fused. The skeleton plasmid (pBHGlox-E1, 3Cre) and GV120/GPR17 siRNA were transfected into HEK293 cells using Lipofectamine 2000 (Invitrogen, Carlsbad, CA, USA). Following the first virus collection, second transfection, second virus collection, SOURSE 15Q ion exchange purification, and filtration, the purified virus was obtained. The transfection efficacy was confirmed by detecting the virus titer.

### Animals and treatments

**Figure 1.**
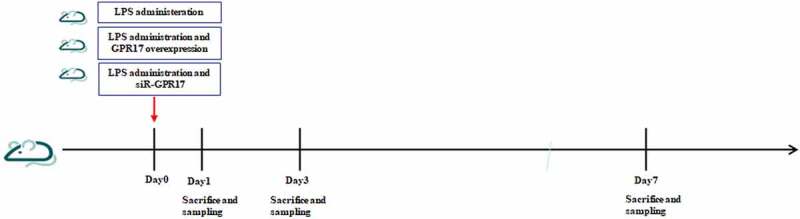
Schedule of animal experiments

The 2-day old rats were obtained from the Beijing Vital River Laboratory Animal Technology Co., Ltd. and anesthetized using diethyl ether. LPS was administered slowly into one side of the brain using a brain stereo locator and microinjection pump to establish the PVL rat model [18]. The animals were divided into five groups: sham, PVL, negative control (NC), GPR17-overexpressed, and siR-GPR17 groups. Animals in the sham group were administered an equal volume of sterilized PBS buffer on one side of the brain. The rats in the NC and GPR17-overexpressed group were transfected with a blank plasmid and pDC315/GPR17, respectively, at the same time as injection of LPS. The animals in the siR-GPR17 group were transfected with siRNA against GPR17 at the same time as LPS injection. The number of animals in each group was 8. All animals were injected with BrdU immediately after the modeling and euthanized at 24 h, 3 days, and 7 days post-modeling for detection. The arrangement of the animal experiments is shown in Figure 1.

### *Isolation of OPCs*[19]

OPCs were isolated from animals in the sham, PVL, GPR17-overexpressed, and siR-GPR17 groups. In brief, brain tissues were isolated and placed in HBSS buffer, followed by removal of the olfactory bulb and separation of the cerebral hemispheres. The brain membrane was stripped, the hippocampus and thalamus were removed, and the remaining cortex was placed in the culture medium. Each cortex was cut into 2–3 pieces, followed by transfer into neurobasal medium (Gibco, California, USA). After blowing for 10 times to separate the cortex until a rare block mass was observed, the sample was centrifuged at 800 rpm for 5 min and the supernatant was removed, followed by resuspension with the neurobasal medium to obtain a single-cell suspension. After incubation using the neurobasal medium for 10 days, the medium was changed to OPC medium (DMEM, 4 mM L-glutamine, 1 mM sodium pyruvate, 0.1% BSA, 50 µg/ml Apo-transferrin, 5 µg/ml insulin, 30 nM sodium selenite, 10 nM D-biotin, 10 nM hydrocortisone, 10 ng/ml PDGF-AA, and 10 ng/ml b-FGF), and cells were cultured in 6-well plates pre-loaded with 1× Poly-D and L-ornithine. Finally, OPCs were obtained.

### *Wound healing assay*[20]

OPCs were grown to confluency and a linear wound was made by scraping a non-opening Pasteur pipette across the confluent cell layer. Cells were washed twice to remove detached cells and debris, followed by incubation for 48 h. The cells migrated into the wound surface, which was considered as the process of in vitro healing. The wound healing in vitro was photographed using an inverted fluorescence microscope and assessed in terms of rate of closure. The rate of wound healing = [(wound width of 0–48 h)/0 h wound width] × 100%.

### *Real-time PCR analysis*[21]

Total mRNA was extracted from lung tissues with TRIzol (Invitrogen, USA), and mRNA was then transcribed to cDNA using the PrimeScript TM RT reagent kit (TAKARA Bio Inc., Kusatsu, Japan). The relative mRNA expression levels of cytokines were determined using an ABI 7500 Fast Real-Time PCR System (Thermo Fisher Scientific, USA). The GAPDH gene was used as an internal standard gene, and the 2^−ΔΔCT^ method was utilized to quantitatively analyze the data. The sequences were as follows: GAPDH: sense: 5ʹ-GGCAAGTTCAATGGCACAGT-3ʹ, anti-sense: 5ʹ-TGGTGAAGACGCCAGTAGACTC-3ʹ; GPR7: sense: 5ʹ-CTGCTACCTGCTGATCATTCG-3ʹ, anti-sense: 5ʹ-TAGACTGAACGGTGGATGTGG-3ʹ; Olig1: sense: 5ʹ-CAGATGTACTATGCGGTTTC-3ʹ, and 5ʹ-AGGATGACGAGATGGGTG-3ʹ.

### *Western blotting assay*[22]

Tissue lysis buffer (Thermo, Massachusetts, USA) was used to isolate total proteins from the brain tissues, which were quantified using the BCA kit (Beyotime, Shanghai, China). Approximately 40 μg of protein from each sample was loaded and separated using SDS-PAGE and transferred onto a PVDF membrane (Thermo, Massachusetts, USA), which was incubated with primary antibodies against GPR17 (CST, 1:1000, Boston, USA), Olig1 (CST, 1:1000, Boston, USA) or β-actin (CST, 1:1000, Boston, USA) at 4°C overnight. After being washed three times with TBST buffer, the membrane was incubated with anti-mouse secondary antibodies (CST, 1:1000, Boston, USA) for 1 h at room temperature, followed by the addition of ELC solution and exposure to Tanon 5200 (Tanon, Beijing, China). ImageJ software was used to quantify the bands.

### *Observation of the myelin sheath in white matter using a transmission electron microscope (TEM)*[23]

Brain tissues were fixed with osmium tetroxide (OsO4) for 90 min, followed by washing with DI water. Subsequently, the tissues were dehydrated with a series of ethanol grades (50%, 70%, and 90%), followed by embedding in epoxy resin and cutting into ultra-thin sections (50–60 nm). Finally, the sections were double-stained with 3% uranyl acetate and lead citrate, then viewed using a JEM 1230 TEM (JEOL, Tokyo, Japan).

### Statistical analysis

The experimental data were statistically analyzed using the Mann-Whitney U-test, and statistically significant differences were reported if P < 0.05. Data are reported as mean ± SD from at least three separate experiments.

## Ethic statements

All animal experiments were carried out in accordance with the National Institutes of Health Guide for the Care and Use of Laboratory Animals (NIH Publications No. 8023, revised 1978), and were approved by the Animal Ethics Committee of Guangdong Provincial Academy of Chinese Medical Sciences (No. 2019033).

## Results

We hypothesized that GPR17 was involved in the pathogenesis of PVL, and that the regulatory effect of GPR17 was associated with the expression level of Olig1. We first determined the expression changes of GPR17 and Olig1 in rats on days 1, 3, and 7 post-PVL modeling, respectively. Subsequently, the expression levels of GPR17 and Olig1 in rats were measured in the sham, PVL, NC, GPR17-overexpressed, and siR-GPR17 groups on days 1, 3, and 7 after PVL modeling, respectively, accompanied by observation of myelin regeneration and repair after PVL white matter injury using TEM. Lastly, OPCs were isolated from each animal to evaluate their migration ability and the expression levels of GPR17 and Olig1.

### GPR17 was down-regulated and Olig1 was up-regulated as time increased in GPR17 knock-down PVL rats

**Figure 2.**
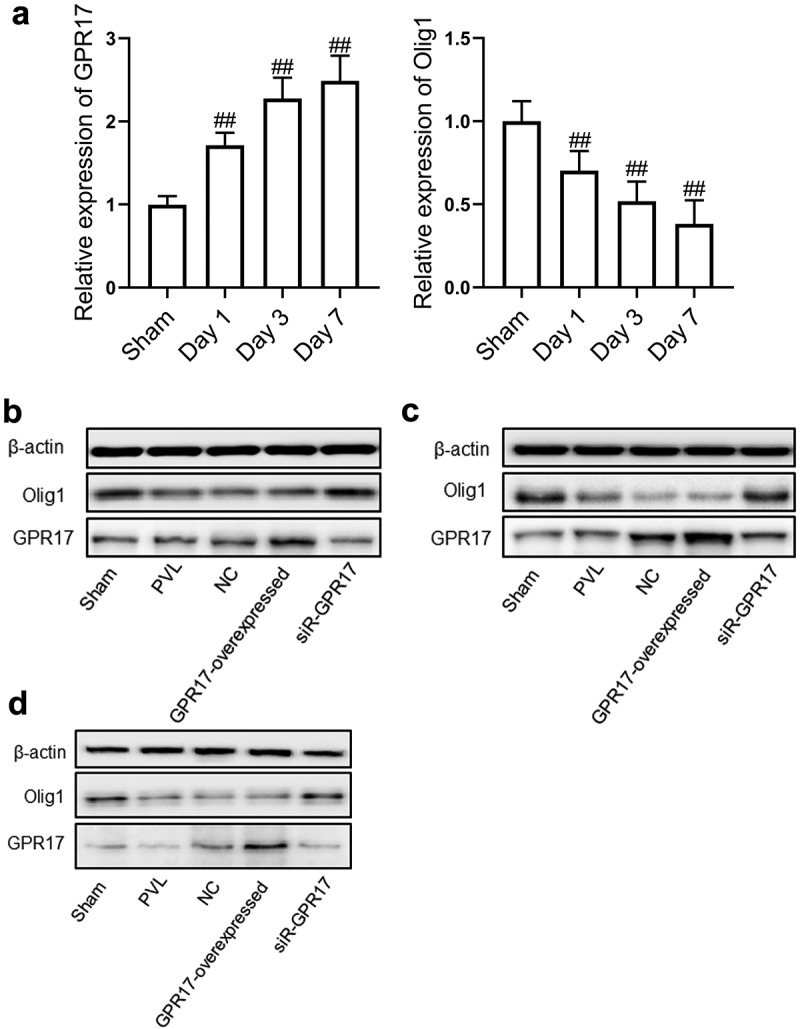
The expression level of GPR17 and Olig1 in PVL rats was negatively related. a. The gene expression level of GPR17 and Olig1 was detected by RT-PCR (##p < 0.01 vs. sham). b. The expression level of GPR17 and Olig1 in PVL rats on Day 1 post-modeling was determined by Western blotting. c. The expression level of GPR17 and Olig1 in PVL rats on Day 3 post-modeling was determined by Western blotting. d. The expression level of GPR17 and Olig1 in PVL rats on Day 7 post-modeling was determined by Western blotting

To investigate the expression changes of GPR17 and Olig1 in the brain tissues following different treatment strategies, RT-PCR and Western blotting assays were performed. The results of RT-PCR were shown in Figure 2a. Compared to the Sham group, the expression level of GPR17 was significantly elevated and the expression level of Olig1 dramatically declined in a time-dependent manner in the PVL group (##p < 0.05 vs. Sham).

As shown in Figure 2b, compared to the sham group, 1 d after modeling, no significant difference was observed in the expression level of GPR17 in the PVL and NC groups. However, Olig1 was significantly downregulated in the PVL and NC groups. Compared to the NC group, GPR17 was significantly upregulated, while Olig1 was downregulated in the GPR17-overexpressed group. In the siR-GPR17 group, the expression of GPR17 was dramatically suppressed and the expression of Olig1 was greatly elevated.

As shown in Figures 2c, 3 days after modeling, compared to the sham group, the expression level of GPR17 was significantly elevated, while that of Olig1 was greatly decreased in the PVL and NC groups. Compared to the NC group, GPR17 was dramatically upregulated and Olig1 was downregulated in the GPR17-overexpressed group. In addition, GPR17 expression was dramatically suppressed, and the expression of Olig1 was greatly increased in the siR-GPR17 group.

**Figure 3.**
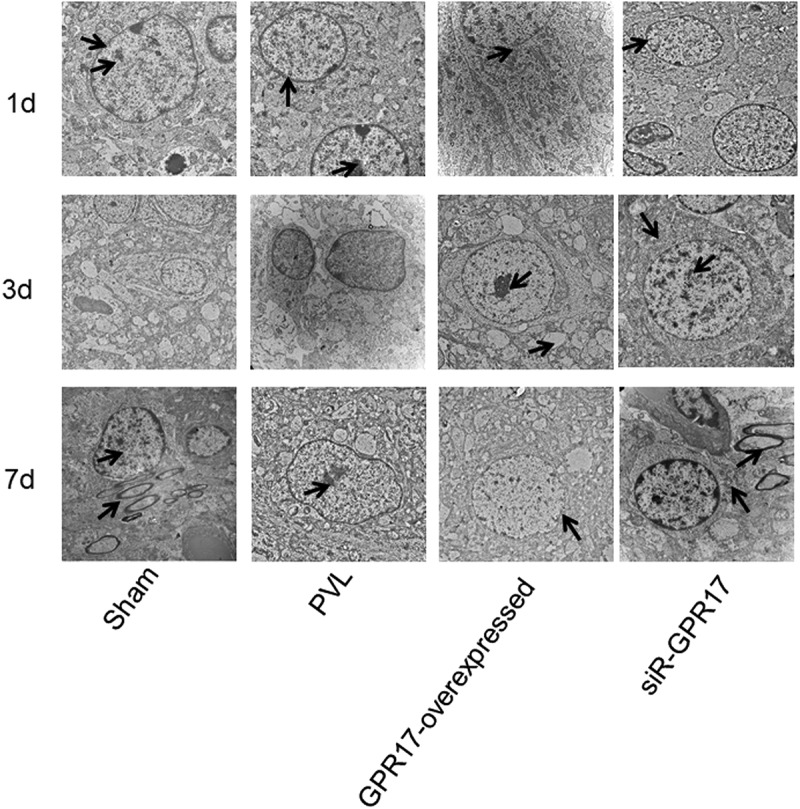
The knockdown of GPR17 contributed to the repair and regeneration of myelin sheath post-PVL. The ultrastructure of brain tissue isolated from each animal was visualized by TEM

As shown in Figure 2d, compared to the sham group, 7 days after modeling, the expression of both GPR17 and Olig1 was markedly inhibited in the PVL group. GPR17 was significantly upregulated, while Olig1 was dramatically downregulated in the NC group. Compared to the NC group, GPR17 was found to be upregulated, while Olig1 was downregulated in the GPR17-overexpressed group. The expression of GPR17 was greatly suppressed, while the expression of Olig1 was greatly elevated in the siR-GPR17 group. In addition, compared to 24 h post-modeling, the change in GPR17 and Olig1 expression at 7 days post-modeling was more significant.

### GPR17 protected neurons by inducing myelin regeneration and repair after PVL white matter injury

We further investigated the effects of GPR17 on the formation of myelin in the white matter of PVL rats. As shown in Figures 3, 24 h post-modeling, in the Sham group, an increased number of bulky neurons, circular nuclei, abundant chromatin within the nucleus, clear nucleoli, rich rough endoplasmic reticulum and ribosomes in the cytoplasm, and small volume mitochondria were observed, as well as fewer astrocytes and high electron densities. The myelin structure developed, and the axoplasm was filled with partial mitochondria and neurofilaments. Part of the axonal membrane was closely connected to the lamellar myelin sheath, and the formation of myelin was fine with a regular shape and a clear boundary. In the PVL and NC groups, small volumes of oligodendrocytes, chromatin condensation, pyknotic nucleoli, and swollen mitochondria were observed. The structure of the partially swollen cytoplasm was open, accompanied by dissolved or degrading/missing organelles. The number of myelin sheaths between cells decreased, and the structure of myelin lamina was unclear, and it was loosely arranged. In the GPR17-overexpressed group, no significant changes were observed compared to the NC group. In the siR-GPR17 group, the cellular structure was fine, with mitochondria slightly swollen in the cytoplasm, as well as vague cristae. Large-volume neurons with round nuclei were observed, and the euchromatin in the nucleus was abundant with less heterochromatin. In gliocytes, apparent mitochondrial swelling was observed with vague cristae and loss of myelin sheaths. The stratification between myelin sheaths was significantly reduced, with a disordered structure.

Three days after modeling, in the sham group, an abundant amount of vascular endothelial cells with ample rough endoplasmic reticulum and ribosomes in the cytoplasm, as well as small volume mitochondria, were observed. The number of astrocytes was relatively small, with high electron density. Some mitochondria and nerve microfilaments were observed in the axoplasm, and some axonal membranes were closely connected to the lamellar myelin sheath. Each axon bundle was wrapped in each neuronal membrane. The nerve fibers were developing, and the cytoplasm located between adjacent mesangial membranes nearly disappeared, which resulted in adhesion between the mesangial membrane cytoplasmic surface. Deep stains were observed at the cross-sections of the nerve fibers. In the PVL and NC groups, necrosis after nuclear pyknosis in many cells was observed in the periventricular lesion. The structure of the brain tissues was loose and soft, with numerous areas of cystic necrosis and voids in the white matter. The myelin structure was loose, and the stratification between myelin sheaths was uneven. In the GPR17-overexpressed group, a small volume of oligodendrocytes, chromatin condensation, pyknotic nucleoli, and swollen mitochondria were observed. The structure of the partially swollen cytoplasm was open and it was accompanied by dissolved or disappearing organelles. The number of myelin sheaths between cells decreased, and the structure of the myelin lamina was unclear and loosely arranged. Compared to the siR-GPR17 group at 1 d post-modeling, no significant changes were observed in the siR-GPR17 group at 3 days post-modeling.

Severn days post-modeling, regular cellular structures and intact cell membrane structures were observed in the oligodendroglial cells in the sham group, with clear nuclear membranes and large nuclei. Chromatin was abundant and evenly distributed throughout the nucleus. The shape of the myelin sheath was clear and the stratification between myelin sheaths was tight. Well-developed axons were observed in the myelin sheaths. In the PVL and NC groups, cellular injuries were apparent, and most neurons were swollen, with low electronic density and small numbers of apoptotic cells. The neuronal nucleus was oval and irregular, and the nuclear chromatin was loose. The mitochondria were severely swollen, and the cristae ruptured and dissolved. The number of astrocytes was relatively small, with a high electronic density. The number of myelin sheaths between cells decreased the structure of the myelin lamina, although it was unclear. The morphology of the myelinated nerve fibers was irregular and loosely arranged. Myelin sheath thicknesses were significantly reduced. Axons were swollen, some mitochondria and nerve microfilaments were observed in the axoplasm, and some axonal membranes were separated from the lamellar myelin sheath. Compared to the GPR17-overexpressed group at 3 days post-modeling, no significant changes were observed in the GPR17-overexpressed group at 7 days post-modeling. In the siR-GPR17 group, a tight junction was observed between the endothelial cells and neurons. The morphology of myelinated nerve fibers was regular, with unambiguous borders and myelin sheaths uniform in thickness and density. The volume of neurons was enlarged with abundant intracytoplasmic organelles. The rough endoplasmic reticulum was arranged in a cord-like pattern, and ribosomes were abundant. Mitochondria were distributed in the cytoplasm in a round or rod-like shape, and the Golgi complex was observed around the nucleus. The volume of oligodendroglial cells significantly decreased. The morphology of the myelinated nerve fibers was regular, and the boundary was clear.

These TEM results indicated that the neurons could be protected by downregulating GPR17, which further contributed to myelin regeneration and repair after white matter injury during PVL.

### GPR17 suppressed the migration of OPCs by downregulating Olig1

**Figure 4.**
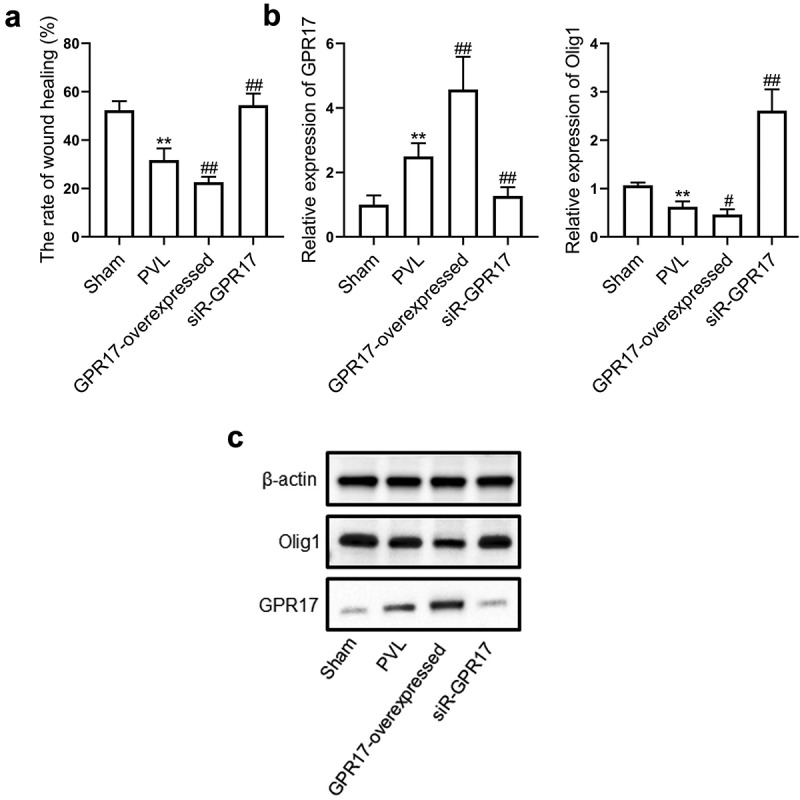
GPR17 suppressed the migration of OPCs by downregulating Olig1. a. Their migration ability was evaluated by a wound healing assay. b. The gene expression levels of GPR17 and Olig1 were determined by RT-PCR. c. The protein expression levels of GPR17 and Olig1 were measured by Western blotting (**p < 0.01 vs. sham, #p < 0.05 vs. PVL, ##p < 0.01 vs. PVL)

To further verify the function of GPR17 in the development of PVL, OPCs were isolated from sham, PVL, GPR17-overexpressed, and siR-GPR17 groups, respectively. A wound healing assay was conducted on these OPCs to evaluate their migration ability. As shown in Figure 4a, compared to the sham group, the wound healing rate was significantly lower in the PVL group, which was further decreased in the GPR17-overexpressed group and significantly elevated in the siR-GPR17 group (**p < 0.01 vs. sham, ##p < 0.01 vs. PVL). RT-PCR and Western blotting assays were used to determine the expression levels of GPR17 and Olig1 in isolated OPCs. As shown in Figure 4b-c, compared to the sham group, GPR17 was upregulated while Olig1 was downregulated in the PVL group, which were further aggravated in the GPR17-overexpressed group and reversed in the siR-GPR17 group (**p < 0.01 vs. sham, #p < 0.05, vs. PVL, ##p < 0.01 vs. PVL). These data indicated that GPR17 downregulated Olig1, thus inhibiting the migration of OPCs in PVL animals.

## Discussion

PVL is the most severe clinical symptom of white matter damage in premature infants. In developing countries, the morbidity of PVL is approximately 8%–26%, among which 66% patients suffer from brain paralysis [24]. In developed countries, 10% of premature infants finally develop brain paralysis, and 25%–50% premature infants experience cognitive disorders, behavioral deficits, and mild motor impairments [25]. Therefore, PVL has become the most common disease to severely affect the long-term quality of life of premature infants. Although the pathological mechanism underlying PVL is unclear, it is reported that abnormal secretion of inflammatory factors, oxidative injury induced by reactive oxygen species (ROS), and hypoxic ischemia are involved in the pathogenesis of PVL [26–28]. According to the investigations of Craig et al [29]., neural development is immature in 2-5-day newborn rats, which is equivalent to 28 to 32 weeks of human gestation and consistent with the time window when white matter damage occurs during the human perinatal period. Therefore, in the present study, 2-day-old newborn rats were used to simulate the clinical characteristics of human PVL. According to the ultrastructure of brain tissues, as time progressed (1 d post modeling to 7 days post modeling), dynamic progression of healthy neural development, formation of myelin sheaths, and development of axons were observed in the brain tissues of animals in the sham group. However, in the PVL group, gradually aggravated neural damage, degradation of myelin sheaths, and separation of axonal membranes and lamellar myelin sheaths were observed, indicating that the normal development of white matter was significantly disrupted by PVL modeling. Thus, we concluded that establishment of a PVL model in rats in the current study was successful.

Ischemic hypoxia not only suppresses the normal development of oligodendroglial cells, but also contributes to the downregulation of Olig1 [30,31]. French et al. reported that the expression level of Olig1 was significantly decreased by oxidative stress induced by ischemic hypoxia, which further prevented the differentiation of neural stem cells into oligodendroglial cells [31]. Additionally, in multiple sclerosis, the repair and regeneration of the myelin sheath can be facilitated by the nuclear transcription function of Olig1 [32]. Olig1 localizes in the nucleus of oligodendrocyte precursors during the embryonic period, and it is later translocated to the cytoplasm postnatally [33]. However, when demyelination injury occurs, Olig1 is translocated into the nucleus and triggers the transcription of related factors to repair and regenerate the myelin sheath [33]. GPR17 has recently been regarded as a sensor of brain injury, and is possibly involved in the regulation of endogenous repair of brain injury [34]. Although the function of GPR17 in the development of PVL has been partly reported [35], the relationship between the expression level of GPR17 and dynamic pathological changes in brain tissues, as well as the relationship between the expression levels of GPR17 and Olig1, have not been elucidated yet. In the present study, we found that in the PVL animals, as time progressed (1 d post modeling to 7 days post modeling), GPR17 was upregulated and Olig1 was downregulated in a time-dependent manner. In addition, the expression level of Olig1 was significantly elevated in the GPR17 knockdown group while the expression level of Olig1 was greatly suppressed in the GPR17-overexpressed group, indicating a negative correlation between the expression levels of GPR17 and Olig1. Finally, taking the results of Western blotting and TEM together, we found that the knockdown of GPR17 and the elevated expression of Olig1 were accompanied by the repair and regeneration of the myelin sheath, while the overexpression of GPR17 and the downregulation of Olig1 were accompanied by the degradation of the myelin sheath. These data indicated that GPR17 might induce the development of PVL, and Olig1 might suppress the development of PVL. However, the mechanism underlying the interaction between GPR17 and Olig1 remains unclear. In our future work, the regulatory mechanism of GPR17 on the expression levels of Olig1 will be investigated to further explore the pathogenesis of PVL.

## Conclusion

Our results revealed that the regeneration and repair of myelin sheath post-PVL white matter injury were induced by downregulating the GPR17 gene, which elevated the expression of Olig1.

## Highlights

GPR17 is a pathogenic gene of PVL

Knockdown of GRP17 regulates myelin sheaths

GRP17 interacts with Olig1
